# Spatial Analysis of the Neighborhood Risk Factors for Respiratory Health in the Australian Capital Territory (ACT): Implications for Emergency Planning

**DOI:** 10.3390/ijerph17176396

**Published:** 2020-09-02

**Authors:** Sarah Davies, Paul Konings, Aparna Lal

**Affiliations:** 1 École des Haute Études en Santé Publique (EHESP), 35043 Rennes, France; sarah.davies@eleve.ehesp.fr; 2 National Centre for Geographic Resources & Analysis in in Primary Health Care (GRAPHC), Canberra 2601, Australia; paul.konings@anu.edu.au; 3 Research School of Population Health, Australian National University, Canberra 2601, Australia

**Keywords:** respiratory health, spatial analysis, health services, environmental health, planning

## Abstract

The Australian Capital Territory (ACT) experienced the worst air quality in the world for several consecutive days following the 2019–2020 Australian bushfires. With a focus on asthma and Chronic Obstructive Pulmonary Disease (COPD), this retrospective study examined the neighborhood-level risk factors for these diseases from 2011 to 2013, including household distance to hospital emergency departments (ED) and general practices (GP) and area-level socioeconomic disadvantage and demographic characteristics at a high spatial resolution. Poisson and Geographically Weighted Poisson Regression (GWR) were compared to examine the need for spatially explicit models. GWR performed significantly better, with rates of both respiratory diseases positively associated with area-level socioeconomic disadvantage. Asthma rates were positively associated with increasing distance from a hospital. Increasing distance to GP was not associated with asthma or COPD rates. These results suggest that respiratory health improvements could be made by prioritizing areas of socioeconomic disadvantage. The ACT has a relatively high density of GP that is geographically well spaced. This distribution of GP could be leveraged to improve emergency response planning in the future.

## 1. Introduction

### 1.1. Changing Climate, Increasing Health Risk

Climate change and public health are inextricably linked [1]. The increasing exposure to bushfires is one area where this intersection occurs as recognized by its addition as an indicator followed globally and in Australia since 2019 in the Lancet Countdown: Tracking Progress on Health and Climate Change, and the planned future expansion of this indicator to include exposure to bushfire smoke [2,3]. Globally, 152 of 196 countries showed increases in population exposure to wildfires during 2015–2018 when compared with 2001–2004 [3]. In Australia, the same comparison showed a slight increase in 2015–2018 but represented substantial adverse exposure in both periods. It is noted that inter-annual climate drivers, the El Niño–Southern Oscillation, the Indian Ocean Dipole, and the Southern Annular Mode, affect the interpretation of yearly comparisons in Australia [2].

The evidence from observations and climate modelling in Australia reveal long-term warming trends, an increase in the frequency of extreme heat events, reduced rainfall and more time in drought in southern Australia, all of which are projected to continue into the future. These changes have contributed to a long-term increase in extreme fire weather and have extended the length of the fire season across large parts of Australia [4,5].

The impact of these changing trends was witnessed in the 2019–2020 Australian bushfire season, which was unprecedented in its extent and severity with its full effects yet to be completely assessed. A recently published preliminary evaluation of the health burden resulting from this event across southeastern Australia has attributed 31 excess deaths, 147 hospital respiratory admissions, 82 cardiovascular hospital admissions, and 89 emergency department attendances for asthma exacerbations to the exposure [6]. Associations between bushfire smoke exposure and respiratory health effects have also been well documented in other locations [7,8].

Two of the worst disasters in Australian Capital Territory (ACT) history have been the result of bushfires during periods of prolonged drought in 2003 and 2019–2020. In 2003, four people died and approximately 500 homes were destroyed in the southern suburbs of Canberra [9]. From the end of November 2019 through January to early February 2020, the ACT was severely affected by bushfire smoke as a result of fires that burnt out more than 10 million hectares in southern Australia [10]. During this period, residents suffered prolonged exposures to hazardous air quality. PM_2.5_ levels reached a peak on January 1st with 24-h rolling average readings of over 1250 μg/m^3^ compared to the Australian national standard and WHO guideline value of 25 μg/m^3^ and considered to be 25 times above hazardous levels [11,12,13].

### 1.2. Health Planning and Preparedness

As fire seasons lengthen and overlap more between the northern and southern hemispheres and with the frequency of other extreme weather events—heatwaves, floods, cyclones, severe storms—also predicted to increase, this puts a high demand on emergency services, including health services, and will likely require changes in the way resources have traditionally been shared around Australia and between countries in times of disaster response and recovery.

It is the view of many Australian health professionals that the 2019–2020 bushfires exposed a number of weaknesses in Australia’s health system and shortcomings in the planning and coordination to deal with a disaster on this scale [14]. Primary health-care systems came under unprecedented strain and a lack of preparation was felt to particularly affect regional areas where supplies ran short and General practices (GP), often directly affected themselves by the disaster, were continuing to work long hours without assistance. The length of time that major cities were exposed to bushfire smoke, many times over the hazardous limits, was unexpected and doctors felt ill-prepared to provide meaningful advice to their patients. Both the president of the Australian Medical Association (AMA) and the immediate past president of the Australian College of Rural and Remote Medicine identified a need for general practices to be involved in future disaster and emergency planning, citing different responsibilities for services between federal and state and territory governments as one underlying reason for a lack of coordination [14].

### 1.3. Respiratory Health

Asthma is a common chronic inflammatory condition of the airways causing episodes of wheezing, breathlessness, and chest tightness which vary in their severity. Australia has one of the highest prevalence rates of asthma in the world [15], with 11.2% of the population affected in 2018 following an increasing trend over the previous 10 years [16]. The exact causes of asthma in individuals are not well understood, but evidence points to a complex interplay of genetic and environmental factors where probable increased risks of developing asthma are associated with exposure to outdoor air pollution, airborne sensitizers or irritants in the workplace or home, molds, certain allergens, ryegrass pollens, and cigarette smoke. In addition, there is also evidence of probable increased asthma risk associated with maternal obesity, smoking, and low vitamin D levels as well as low birthweight, childhood obesity, and exposure to paracetamol, antibiotics, proton pump inhibitors, and H_2_ receptor antagonists in childhood [17].

Chronic Obstructive Pulmonary Disease (COPD) is a progressive inflammatory lung disease that limits airflow to the lungs. It mainly affects older people and, although treatment can control symptoms and slow progression, it is not fully reversible. Around 1 in 20 Australians aged 45 years and over had COPD in 2017–2018 [18]. In 2015, COPD was the third leading specific cause of total disease burden in Australia, and hospitalization rates for the condition have been increasing since 2013–2014 [19]. The primary cause of COPD is cigarette smoking; however, risk factors for non-smokers have also been identified, including exposure to smoke from burning biomass fuels and outdoor air pollution, occupational exposures to dusts and chemicals, treated pulmonary tuberculosis, lower respiratory tract infections during childhood and chronic asthma, poor socioeconomic status, poor nutrition, and poor educational attainment [20]. Acute exacerbations of COPD are often due to respiratory infections but are also associated with increased exposure to air pollutants and changes in temperature [21].

The main aim of this study was to examine, at a high resolution, the spatial relationships between respiratory disease rates and distance to hospital emergency departments (ED) and GP in the ACT using data from the Australian Health Survey from 2011 to 2013. This type of analysis can improve understanding of local area risk factors, inform the targeted prioritization of health resources, and engage health services in emergency planning and preparedness. Addressing local area risk factors through health service planning to improve access has the potential to mitigate the effects of future bushfire events, which, depending on location and weather conditions, will have varying effects on air quality across the ACT.

## 2. Materials and Methods

### 2.1. Study Area

The study area covered all residential areas of the ACT, which is made up for the most part of metropolitan Canberra, but also includes areas more regional in character on its urban fringe. Canberra is centered around the large artificial Lake Burley Griffin and was designed to incorporate significant areas of natural vegetation and be organized around decentralized district town centers rather than one city center. The southern area of the ACT is a large national park without residential areas [22].

### 2.2. Data Sources

Data on rates of respiratory disease, location of health services, residential address locations, and population demographic data were primarily sourced from publicly available Australian government supported datasets as outlined in Table 1. The location of General practices was provided by the Australian National University’s National Centre for Geographic Resources & Analysis in Primary Health Care within the Research School of Population Health based on a commercial marketing database. Residential locations were derived from the Geocoded National Address File (G-NAF) using custom algorithms.

The Australian Bureau of Statistics (ABS) conducts a national census every 5 years with the most recent taking place in 2016. As part of this process, the ABS defines the Australian Statistical Geography Standard (ASGS), consisting of different levels of spatially aggregated data. Where possible, data were collected directly at Statistical Area Level 1 (SA1), which has been designed as the smallest unit for the release of census data. In this study, there were 1013 SA1s, after removing those with a population less than 50 persons. However, some data were only available at the larger SA2 level. The average population of SA2s is approximately 10,000 persons. In this study, there were 103 SA2s. SA1 blocks generally have a population of 200 to 800 persons, an average population of about 400 persons, and are designed to be predominantly rural or urban in character. Variables for the analysis were all consolidated at the SA1 level, as described in Table 1.

### 2.3. Data Exclusions

**Figure 1:**
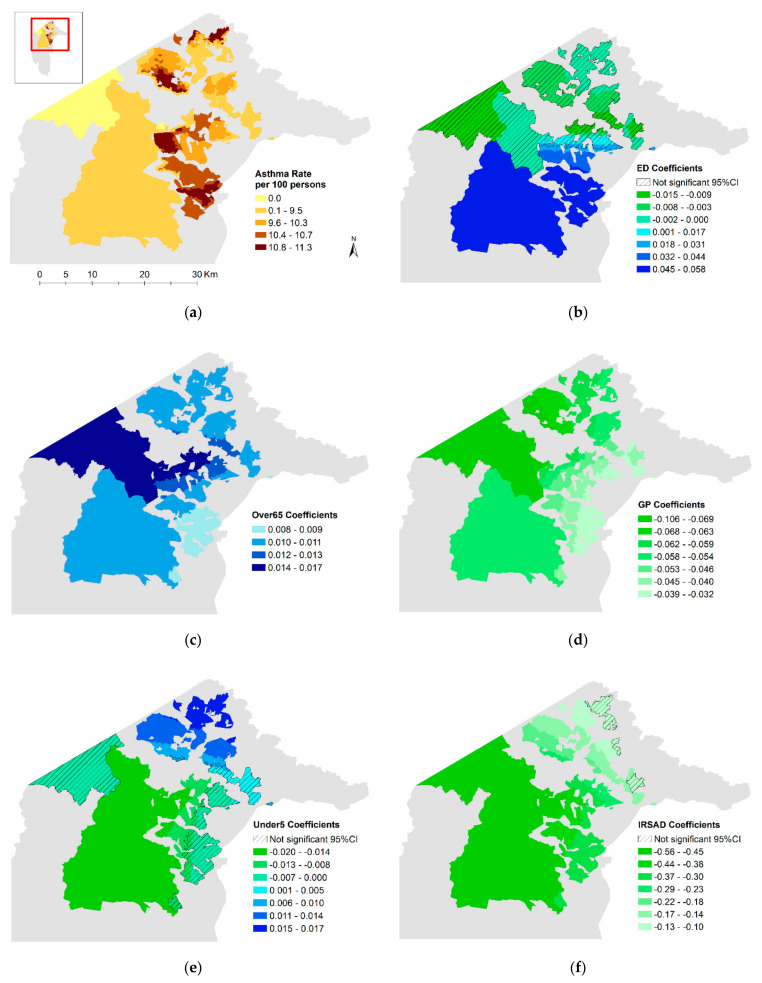

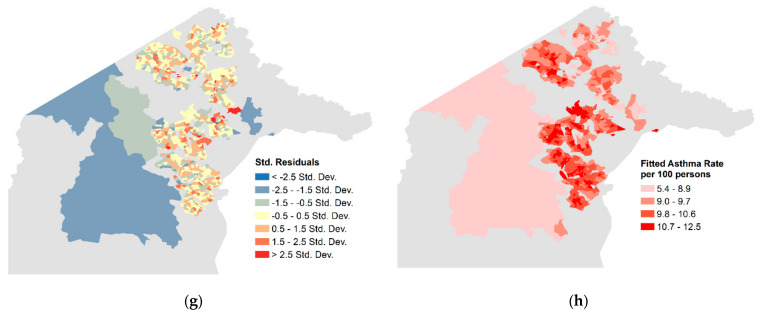
Maps of asthma rate and Geographically Weighted Poisson Regression (GWR) results for residential areas in the ACT: (**a**) Asthma rate; (**b**) ED coefficients; (**c**) Over65 coefficients; (**d**) GP coefficients; (**e**) Under5 coefficients; (**f**) IRSAD coefficients; (**g**) Std. residuals; (**h**) Fitted rate.

**Figure 2:**
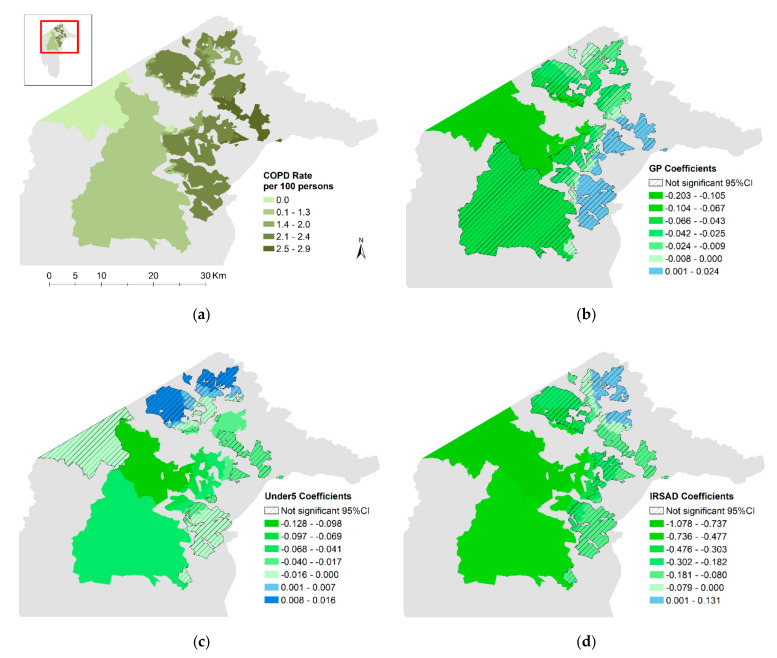

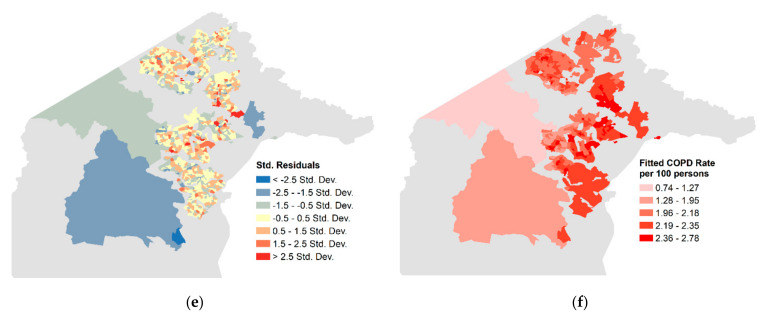
Maps of COPD rate and GWR results for residential areas in the ACT: (**a**) COPD rate; (**b**) GP coefficients; (**c**) Under5 coefficients; (**d**) IRSAD coefficients; (**e**) Std. residuals; (**f**) Fitted rate.

There were 1147 SA1s defined by the ABS in the ACT in 2016; however, only 1013 residential blocks were used in the analysis. Nonresidential areas and areas with populations of less than 50 people were excluded from further analysis. This is in line with ABS methods, which do not calculate socioeconomic indicators for areas with small populations, as the results are considered unreliable. The 134 blocks excluded were primarily nature reserves, with other examples being blocks containing the airport, large portions of Lake Burley Griffin, large shopping malls, and sports stadiums. These areas are shown as grey in the figures that follow as part of the analysis (Figure 1 and Figure 2).

### 2.4. Modelling Methods

The association between the prevalence of respiratory disease and access to halth services, as well as sociodemographic factors, were first modelled using Poisson regression, a method commonly used for modelling disease count data. Two chronic respiratory diseases, asthma and COPD, were modelled separately, and the variables used as detailed in Table 1. The choice of independent variables for distance to health services and the composite socioeconomic index IRSAD was informed by a review of literature associating these and similar measures with health service access and asthma and COPD prevalence and exacerbation outcomes [29,30,31,32]. Age data were included for age groups identified as at higher risk of asthma and COPD, that is, over 65 and under 5 years old [19,33]. Log transformations of the variables ED (distance to nearest hospital Emergency Department) and GP (distance to nearest general practice) were used in the analysis as these variables had a wide range and a highly skewed distribution. Histograms of these variables are included in the Supplementary Materials. The variable IRSAD was normalized to bring it onto a similar scale to the other variables. Univariate analysis was performed to assess the association of all variables with respiratory disease counts and those variables with a p value < 0.2 were retained for testing in the multivariate model. The final multivariate models were chosen based on backward selection of variables using likelihood ratio tests to compare models with a p value threshold of 0.05 and are presented in Equations (1) and (2) below. Total population in each SA1 block was included as the exposure variable.

log(Asthma count) = β_0_ + β_1_ ED + β_2_ GP + β_3_ Under5 + β_4_ Over65 + β_5_ IRSAD + log(Pop) + ε (1)

log(COPD count) = β_0_ + β_1_ GP + β_2_ Under5 + β_3_ IRSAD + log(Pop) + ε (2)

where β_0_ is the global intercept, β_k_ (k = 1, 2, 3, 4, 5) are the model regression coefficients corresponding to the adjacent explanatory variable, and ε is the error term.

Once these ’global’ models were defined, the same covariates were used in Geographically Weighted Poisson Regression (GWR) models to examine how coefficients may vary with location and what effect geographic clustering of explanatory variables might have on model results, as shown in Equations (3) and (4). Based on the centroid points of SA1 blocks (u_i_, v_i_), the GWR ran individual regression models for each block, incorporating the dependent and explanatory variables of surrounding blocks falling within a specified distance (bandwidth).

log(Asthma count_i_) = β_0_ (u_i_, v_i_) + β_1_ (u_i_, v_i_) ED_i_ + + β_2_ (u_i_, v_i_) GP_i_ + β_3_ (u_i_, v_i_) Under5_i_ + β_4_ (u_i_, v_i_) Over65_i_ + β_5_ (u_i_, v_i_) IRSAD_i_ + log(Pop_i_) + ε_i_ (3)

log(COPD count_i_) = β_0_ (u_i_, v_i_) + β_1_ (u_i_, v_i_) GP_i_ + β_2_ (u_i_, v_i_) Under5_i_ + β_3_ (u_i_, v_i_) IRSAD_i_ + log(Pop_i_) + ε_i_ (4)

where i represents an individual SA1 block, β_0_ is the regression equation intercept for block i where (u_i_, v_i_) denotes the 2 dimensional coordinates of the centroid of block i, β_k_ (k = 1, 2, 3, 4, 5) are the model regression coefficients corresponding to the adjacent explanatory variable for block i, and ε_i_ is the error term for block i.

The GWR was run with an adaptive bandwidth based on a constant number of neighbors optimized for the lowest corrected Akaike Information Criterion value (AICc).

The assumption that spatial processes are influencing the distribution of variables was also tested by calculating the Global Moran’s Index and Getis-Ord General G statistic for each variable. These tests were run using a spatial weights matrix generated to ensure that all blocks had a minimum of 8 neighbors or otherwise used a fixed-distance band where clustering was found to be most pronounced, determined through incremental spatial autocorrelation. Conceptualization of spatial relationships was set at inverse distance, and no row standardization was used due to the statistical construction of the SA1 blocks.

Statistical analysis and mapping were performed using R version 3.6.3 (R Foundation for Statistical Computing, Vienna, Austria) and ArcMap version 10.7.1 (Environmental Systems Research Institute, Redlands, USA). GWR results were also cross-checked using GWR4 software version 4.0.90 (National Centre for Geocomputation, National University of Ireland Maynooth and Department of Geography, Ritsumeikan University, Japan).

## 3. Results

### 3.1. Spatial Distribution of Variables

Summary information for asthma and COPD rates and the explanatory variables used in modelling are presented in Table 2. The average asthma prevalence rate in the ACT was 9.7 cases per 100 persons. The asthma rate varied across the ACT (Figure 1a) from 0 to 11.3 cases per 100 persons, with lower rates observed in the southern regional areas. Higher rates were observed in southern, central, and northern suburbs of metropolitan Canberra. COPD rates averaged 2.2 cases per 100 persons, ranging from 0 to 2.9. Similar to asthma, low COPD rates were evident in the southern regional areas; however, the spatial variation through metropolitan Canberra was different, with the highest COPD rates observed in the eastern suburbs (Figure 2a).

Initial visual inspection of the spatial distributions of the explanatory variables considered in this study did not point to any strong and consistent correlation in patterns between variables. The average distance to the nearest 24-h hospital emergency department ranged from 0.4 km to 18.4 km. With only 2 hospitals offering this service in the ACT, located in central north and central south Canberra, respectively, those in central suburbs were closer than those in outer suburbs and regional areas. In contrast, the distance to GP was within a fairly small range across urban areas though greater distances needed to be travelled by those on the urban fringe and in the newer northern-most suburbs as well as those in the more regional areas. The average straight-line distance across all areas was 0.8 km and ranged from 0.1 to 11.4 km. The average percentage of the population over 65 years of age by SA1 block was 12.4% with a maximum of 27.2%, while the percentage of the population under 5 years averaged 7.1% with a maximum of 14.5%. There was a higher percentage of residents over 65 years of age in the central suburbs of Canberra with a higher percentage of under-5-year-olds in outer suburbs and regional areas. Relatively advantaged areas were found in the city center as well as around the urban fringe and regional areas, with pockets of relative disadvantage observed across other parts of the urban area. The IRSAD scores ranged from 697 to 1239, with an average of 1088. A full set of variable maps for comparison is included in the Supplementary Materials.

### 3.2. Cluster Analysis

Calculation of the Moran’s Index showed positive spatial autocorrelation for all variables, indicating significant nonrandom spatial clustering. The Getis-Ord G statistic test also showed significant spatial clustering of high and/or low values for all variables except the IRSAD score. The z score for IRSAD was negative, indicating that the spatial distribution of high and/or low values is more dispersed than would be expected if the pattern were random. Detailed test results are included in the Supplementary Materials.

### 3.3. Modelling Results

Comparing the results of the stationary global Poisson models with the GWR models further supports the influence of spatial processes (Table 3). For asthma, the AIC improved from 6601 with the global model to 3153 with the GWR model and the percent of deviance explained improved from 12.8% to 27.9%. For COPD, the AIC improved from 4218 to 774, while the percent of deviance explained improved from 6.3% to 43.7%. The Moran’s Index for the regression residuals for both asthma and COPD when moving to the GWR model remained statistically significant; evidence of autocorrelation indicating that some spatial dependencies are still unaccounted for. Figure 1 and Figure 2 show the regional variations for the residuals and local coefficient values for the variables found to be statistically significant in the global models, as well as the variation for the fitted outcomes. Box plots showing the distribution of variable coefficients are also included in the Supplementary Materials.

### 3.4. COPD

For COPD, fewer variables were significant in the global model compared to asthma, those being distance to GP, percentage under 5, and IRSAD score. The global model estimated that for every increase of 2.7 km in average distance to a GP, the COPD rate would decrease by 3.8% (95% CI 0.5–7.1). In the GWR model for the same distance increase, this effect ranged from a decrease of 18.4% to an increase of 2.4%; however, only the decreasing effect was significant in a small number of western regional and urban fringe blocks (Figure 2b).

For every 1% increase in the percentage of the population under 5 years old, the global model estimated a 1% (95% CI 0.02–2.0) decrease in the COPD rate. Although the GWR local model coefficients ranged from a decrease of 12% to an increase of 1.6% in COPD rate, only the stronger decreasing effects found in the central and southern regions were statistically significant within a 95% confidence interval (Figure 2c).

As with the asthma models, a higher IRSAD score was associated with decreased rates of COPD, and the relationship was stronger moving from the southwest to the northeast, with no northern or easterly blocks statistically significant in the COPD GWR model. The global model estimated a decrease of 4.0% (95% CI 0.5–7.3) in the COPD rate for every 100 point increase in the IRSAD score, with the GWR model ranging from a decrease of 18% to 5.4% where significant (Figure 2d).

## 4. Discussion

In the ACT, rates of asthma and COPD varied spatially at high resolution with distance to health services, age, and socioeconomic status, providing insight into local area risks. Asthma rates were positively associated with increased distance from a hospital emergency department, a higher percentage of the population under 5 and over 65 years old, and increased area-level socioeconomic disadvantage. COPD rates were positively associated with area-level socioeconomic disadvantage and inversely related with the percentage of the population under 5 years old.

### 4.1. Access to Health Services

It is well established that greater access to health services improves health outcomes in populations. In this study, two indicators of physical access were used; that is, straight-line distances to the nearest hospital emergency department and to the nearest general practice. However, distance is only one aspect of accessibility. For example, research focused on access to primary care will use multifaceted frameworks such as that proposed by Levesque, Harris, and Russell [34], which includes both characteristics of services: approachability, acceptability, availability and accommodation, affordability, and appropriateness as well as corresponding abilities of people: ability to perceive, ability to seek, ability to reach, ability to pay and ability to engage. A recent study in Australia has shown that despite a universal health care scheme which provides free or low-cost health care access for all Australians, a significant proportion of the population still faced financial and availability barriers to accessing health care, with 27% of Australian adults reporting difficulties with after-hours access and 16% foregoing healthcare due to cost [35]. There is, however, also evidence that distance to services itself can be associated with poorer health outcomes. A systematic review of 108 studies from North America, Western Europe, Australia, and New Zealand found that 77% of studies showed evidence of worse health outcomes the further a patient lived from health facilities [36]. For asthma and COPD specifically in Australia, the overall prevalence is lower in major cities than regional areas of Australia, such as those that exist outside Canberra in the ACT, where travel time to health services is greater [37]. Therefore, it was expected that if distance was acting as a proxy for access, it would be likely that an association would be observed between greater distance and higher rates of disease. This was found to be the case with distance to ED and asthma. However, this relationship was also found to be spatially heterogenous and for most of the urban Canberra area not significant within a 95% confidence interval. As this relationship becomes increasingly important moving to the outer southern suburbs and south/southwestern regional areas, it may imply that, within a certain range, distance does not have much of an influence, but moving further away from the city center into regional areas, increased distance may be a barrier to access that affects health outcomes. To our knowledge, this is the first time in the ACT that distances from individual households to the nearest ED and GP were estimated.

In contrast, rates of both asthma and COPD were inversely associated with increasing distance to the nearest GP. For the most part, the ACT is well served with GP, and the decentralized nature of Canberra’s geographic planning means that GP are relatively well dispersed geographically with the average distance to GP no further than 4.4 km. Although the rate of full-time equivalent GP in the ACT is the lowest of any state or territory in Australia at 88.4 per 100,000 population, in terms of concentration in a geographic area, it is by far the highest, at least 12 times that of any other state or territory [38]. Some suggestion of how well asthma and COPD patients are accessing good quality primary care within GP practices in the ACT may be inferred by looking at comparative hospital admission rates for these conditions for which the areas of the ACT fall in the lowest quartile in Australia [30].

Due to the location and geographic layout of Canberra, it is likely that those areas furthest from GP are closer to forest and grassland areas, which may contribute to the trends seen. Green space has been shown to buffer traffic air pollution, reducing asthma incidence in at least one study in Australian children [39]. A wider review of 14 studies from North America, Europe, and Australia is also suggestive of an association between urban greenspace and allergic respiratory diseases in childhood, although evidence is inconsistent [40]. A recent large cross-sectional study of more than 96,000 people in the UK found that exposure to ambient PM_2.5_ and urbanicity were associated with a higher risk of COPD, while residing in greener areas was associated with lower risk [41]. The spatial variation of the regression coefficients indicates some support for this premise, with stronger influence of the GP distance coefficients found in the western regional and urban fringe areas for asthma and the only significant relationship found in this same area for COPD. These areas border national park, agricultural land, and the lake, and also contain nature reserves within them, as opposed to the southeastern border, which is adjacent to urban areas in the state of NSW. Further work with more thorough information on access measures and proximity to green space is needed to better understand these relationships.

### 4.2. Age

As a progressive disease, COPD mainly affects people over 45 years of age and has higher prevalence in older age groups [19], whereas asthma can be diagnosed at any age and the symptoms for some children disappear in later life. The modelling results for asthma which showed an association of higher rates with increased percentages of both over 65 year olds and children under 5 years old are consistent with Australia-wide data, which shows that the highest prevalence rates for asthma are in the 0–14-year age group for males and in the 65–74-year age group for females [33]. In the asthma GWR model, greater effects were observed in the central areas, with many of these central blocks also corresponding to those areas with the highest percentages of over-65-year-olds in the ACT. This suggests that, in these areas, advancing age may be an important factor. Although the overall relationship with the percentage of under-5-year-olds was positive, significant spatial variations were found. For COPD, a significant negative association was found with the percentage of population under-5-years-old. While there are differences in the distribution of prevalence rates for the two diseases between age groups, these similarities may be due to the peaks for prevalence falling in similar age categories, between 55 and 74 years of age for asthma and 65 and 74 for COPD [19,33].

### 4.3. Socioeconomic Status

The effects of social determinants on health are well established, and many studies confirm associations between higher rates of chronic respiratory diseases and lower socioeconomic status both in Australia and globally [42,43,44,45]. This is consistent with the results from this analysis, which also shows an association between areas of higher relative disadvantage and lower relative advantage and higher rates of both asthma and COPD in the ACT. Australia-wide data show that prevalence rates for both asthma and COPD are higher in areas with relatively higher socioeconomic disadvantage. This relationship holds true for the population as a whole and also when disaggregated by sex with the prevalence of asthma in socioeconomic areas ranked in the lowest quintile compared with areas ranked in the highest quintile being 13% and 10% for males and 16% and 9.9% for females, respectively [33]. The same comparison for COPD shows a difference of 8.0% and 2.6% for males and 6.9% and 3.9% for females [19]. When comparing with the rest of Australia, the ACT is relatively advantaged with only 0.9% of SA1 blocks falling in the lowest quintile and 54% of SA1 blocks in the highest quintile of Australian IRSAD scores [27]. Despite this overall advantage, the spatial association of higher asthma rates with relative disadvantage is still apparent. The spatial variation of coefficients in both the asthma and COPD GWR models also shows a similar gradient with stronger relationships in the southwest. There is a greater influence in the regional areas and in the central area of Canberra where areas of contrasting IRSAD scores are found, and this may contribute to the patterns observed. Areas of higher socioeconomic disadvantage have also been found to be at higher risk for respiratory ED visits during bushfire events in California where modelling PM_2.5_ exposure found the highest effects on ED visits in areas with the lowest median incomes [46]. This further supports the need to ensure adequate health care access in these areas.

### 4.4. Model Fit

When examining the maps of residuals from the asthma and COPD GWR models, it is observed that both models over-predict in the regional south and on the eastern edge of Canberra around the airport (Figure 1g and Figure 2e). Blocks where the models underpredict are scattered throughout the urban areas and do not appear to be clustered together. Where the models fit well and where they fit poorly can potentially provide some insight into variables that are missing from the model [47]. Future consideration of how factors like access to green space and underlying medical conditions vary spatially to influence respiratory disease risk would be useful.

### 4.5. Limitations, Strengths, and Areas for Future Analysis

An important factor linked to respiratory disease that was excluded was air quality. In general, the ACT has good air quality, but it does change seasonally and would also vary based on local geography and prevailing weather conditions. Higher levels of PM_2.5_ have been measured near major roads in Canberra and wood burning for heat in winter can increase outdoor pollution levels and lead to the ACT government instituting no-burn days [48]. Modelling variation in air quality and its impact over time, particularly including seasonal weather and bushfire events, would be an interesting area for future analysis.

In this analysis, age standardized disease rates at the SA2 level were applied to SA1 blocks as this is the highest resolution that these rates were available. Ideally age standardized rates at the SA1 level would have been used. Sex differences may also have been important to consider, given that the prevalence of asthma by age varies between men and women. Among those aged 0–14 years, asthma is more common in boys but, after 25, becomes more common in women. This change is partly due to changes that occur during adolescent development, as well as differences in environmental exposures [33]. There are also differences with COPD being more prevalent in women compared with men in certain age groups [19]. Disease rates are also known to be higher amongst indigenous populations and linked to lifestyle factors such as smoking and obesity. Future work could consider investigating both these additional demographic factors and incorporate smoking and obesity rates.

The asthma and COPD rates used in this analysis are estimates based on an extension of self-reported survey data and, as such, will have a certain variability associated with them [23]. A future extension of this analysis could consider using GP presentations or hospitalization records as additional data sources.

The major strength of this analysis is that it is the first time such a high-resolution spatial analysis has been carried out to identify associations between distance to health services and respiratory health outcomes in the ACT and one of very few conducted in any other major metropolitan areas in Australia. Certainly, the use of individual households to measure distance to health services provides a very detailed view of how distance is associated with health outcomes. The use of geographically weighted regression was able to identify specific parts of the study area where relationships differed from the global model and can guide the development of targeted interventions to protect vulnerable groups.

### 4.6. Implications for Health Service Planning

Not only has analysis of hospital admissions and emergency department attendance data from the 2019–2020 bushfire season confirmed the importance of the short-term effects of prolonged smoke exposure [6], but shorter exposures have also been demonstrated to increase hospital admissions in an analysis of many shorter bushfire events over a 13-year period [49]. With the frequency of bushfire events predicted to increase and the longer-term effects of the unprecedented smoke exposures experienced by those in the ACT in 2019–2020 still unknown, it will be become increasingly important to ensure that health systems are equipped with the knowledge and resources to address these challenges and protect those with increased vulnerabilities, whether due to pre-existing medical conditions, age, or socioeconomic status.

The results of this analysis show that outside the central urban area of Canberra, the risk of asthma and COPD is positively associated with increasing distance from the major hospitals. These more regional areas are also at increased risk of the direct impact of bushfires, and this dimension should be considered in emergency response planning to ensure that adequate health care is available to these areas in times of crisis.

The relatively short distance to the nearest GP from anywhere in the ACT (a maximum of 4.4 km in the metropolitan area and up to 11.4 km outside) would support the calls from GP themselves during the recent catastrophic bushfire season that they take a more formal role in disaster and emergency planning and response. Planning of services is challenging in growing cities and especially in those that will come under increasing threats of natural disasters. The best methods to design urban environments involves an active area of research and debate, and the decentralized design of Canberra with its dispersed GP services, may offer an example in other locations [50].

The positive relationship between lower socioeconomic status and higher asthma and COPD rates within Canberra, despite the overall relative advantage of the ACT, is important. It is interesting to consider this result in the wider discussion of the mechanisms by which neighborhood effects can influence health, not only through physical access to services but through the social stressors and networks that may be operating to aid or inhibit access and influence individual behaviors over both the short and long term [51]. If disadvantaged populations are also located at the urban fringes where they are at a higher risk of losing their homes to bushfires, we need to develop targeted programs and services to address health inequities. Spatial analyses such as these can aid in decision-making for health services so the right populations can be targeted at the right time.

## 5. Conclusions

Spatial analyses can provide important insights into the geographic clustering of populations that are at high risk and the area-level and demographic factors that contribute to this risk. Respiratory health improvements could be made by prioritizing areas of socioeconomic disadvantage. Outside the central urban area of Canberra, the risk of asthma and COPD is positively associated with increasing distance from the major hospitals. These more regional areas are also at heightened risk of bushfires and emergency response planning or these populations should consider access to hospitals. In addition, the relatively small distance of households to GP across the ACT could be leveraged to support emergency care in regional areas and improve planning and response to meet health needs in future emergencies, including bushfires.

## Figures and Tables

**Table 1:** Variables at the SA1 level.

Dependent Variables			
Name	Description	Data Integration	Source
Asthma count	Estimated number of people with asthma prevalent over a 2-year period, 2011–2013	Asthma rate at 2011 SA2 level applied to 2016 SA1 level using a spatial join and averaging the rates for intersecting polygons. Asthma count then calculated as SA1 asthma rate x SA1 population and rounded to nearest whole number	Torrens University Australian Public Health Information Development Unit accessed through the Australian Urban Research Infrastructure Network-AURIN [23]
COPD count	Estimated number of people with COPD prevalent over a 2-year period, 2011–2013	COPD rate at 2011 SA2 level applied to 2016 SA1 level using a spatial join and averaging the rates for intersecting polygons. COPD count then calculated as SA1 asthma rate x SA1 population and rounded to nearest whole number	
**Explanatory Variables**			
**Name**	**Description**	**Data Integration**	**Source(s)**
ED	Average straight-line distance from each residence to nearest hospital emergency department	Euclidian distance between each residence location and nearest ED calculated then averaged over all residences within each SA1 block	Residence locations: Geo-coded National Address File (G-NAF) [24] Hospital locations: ACT Government [25]
GP	Average straight-line distance from each residence to the nearest General practice	Euclidian distance between each residence location and nearest GP calculated, then averaged over all residences within each SA1 block	Residence locations: Geo-coded National Address File (G-NAF) [24] GP locations: Datajet licensed marketing database accessed through the Australian National University Research School of Population Health
Under5	Percentage of the population under 5 years of age	Data at SA2 level applied to each SA1 block within it	Australian Bureau of Statistics 2016 census data [26]
Over65	Percentage of the population over 65 years of age	Data at SA2 level applied to each SA1 block within it	
IRSAD	Index of Relative Socioeconomic Advantage and Disadvantage. Low values indicate a high proportion of relatively disadvantaged people and a low proportion of relatively advantaged people in an area	Data available at SA1 level	Australian Bureau of Statistics 2016 census data [27]
Pop	Total population	Data available at SA1 level	Australian Bureau of Statistics 2016 census data [28]

**Table 2:** Summary statistics of modelling variables.

Variables: N = 1013	Mean	SD	Median	Min	Max
Asthma Rate (per 100 persons)	9.7	1.6	10.1	0	11.3
COPD Rate (per 100 persons)	2.2	0.3	2.3	0	2.9
Asthma Count	37.2	14.1	36.0	0	166
COPD Count	8.3	3.2	8.0	0	37
Distance to ED (km)	6.4	3.0	6.2	0.4	18.4
Distance to GP (km)	0.8	0.6	0.6	0.1	11.4
Population (N)	383	130	368	50	1564
Population under 5 (%)	7.1	2.3	7.1	0	14.5
Population over 65 (%)	12.4	5.8	12.4	0.5	27.2
IRSAD Score	1088	62	1092	697	1239

**Table 3:** Summary of regression results.

Variable	Asthma Count						COPD Count					
	Poisson			GWR-Poisson			Poisson			GWR-Poisson		
	Coeff	Std Error	VIF	Local Coefficients			Coeff	Std Error	VIF	Local Coefficients		
				Mean	Median					Mean	Median	
ED	0.035 **	0.003	1.58	0.014	−0.002							
GP	−0.054 ***	0.008	1.22	−0.054	−0.057		−0.039 *	0.017	1.17	−0.024	−0.020	
Under5	0.006 *	0.003	1.64	0.002	−0.007		−0.010	0.005	1.16	−0.022	−0.007	
Over65	0.010 ***	0.001	1.57	0.011	0.010							
IRSAD	−0.243 ***	0.048	1.08	−0.255	−0.215		−0.221 *	0.098	1.03	−0.224	−0.170	
Morans I residuals	Index	z	*p*	Index	z	*p*	Index	z	*p*	Index	z	*p*
	0.16	49.2	0.0000	0.04	7.5	0.0000	0.179	54.5	0.0000	0.019	6.1	0.0000
Deviance explained	0.128			0.279			0.063			0.437		
AIC	6601			3153			4218			774		

*p* < 0.1 * significant at 95% (*p* < 0.05), ** significant at 99% (*p* < 0.01), *** significant at 99.9% (*p* < 0.001).

## Supplementary Materials

The following are available online at https://www.mdpi.com/1660-4601/17/17/6396/s1, Figure S1: Variable Maps, Figure S2: Histograms of ED distance and natural log transformed ED distance, Figure S3: Histograms of GP distance and natural log transformed GP distance Table S1: Cluster analysis results all variables: Moran’s Index and Getis-Ord General G Statistic, Figure S4: Geographically Weighted Poisson Regression Local Coefficients for Asthma, Figure S5: Geographically Weighted Poisson Regression Local Coefficients for COPD.
