# Supplementary material for: Spatial Analysis of the Neighborhood Risk Factors for Respiratory Health in the Australian Capital Territory (ACT): Implications for Emergency Planning
Source: Int J Environ Res Public Health. 2020 Sep 2;17(17):6396. doi: 10.3390/ijerph17176396 (PMC7503909; doi:10.3390/ijerph17176396)

Supplementary material

Spatial analysis of the neighbourhood risk factors for respiratory health in the Australian Capital Territory (ACT): Implications for emergency planning

Sarah Davies [1], Paul Konings[2] and Aparna Lal [3,*]

[1] École des Haute Études en Santé Publique (EHESP); sarah.davies@eleve.ehesp.fr
[2] National Centre for Geographic Resources & Analysis in in Primary Health Care (GRAPHC); paul.konings@anu.edu.au
[3] Research School of Population Health, Australian National University, Canberra, Australia; aparna.lal@anu.edu.au
* Correspondence: aparna.lal@anu.edu.au

Figure S1: Variable Maps

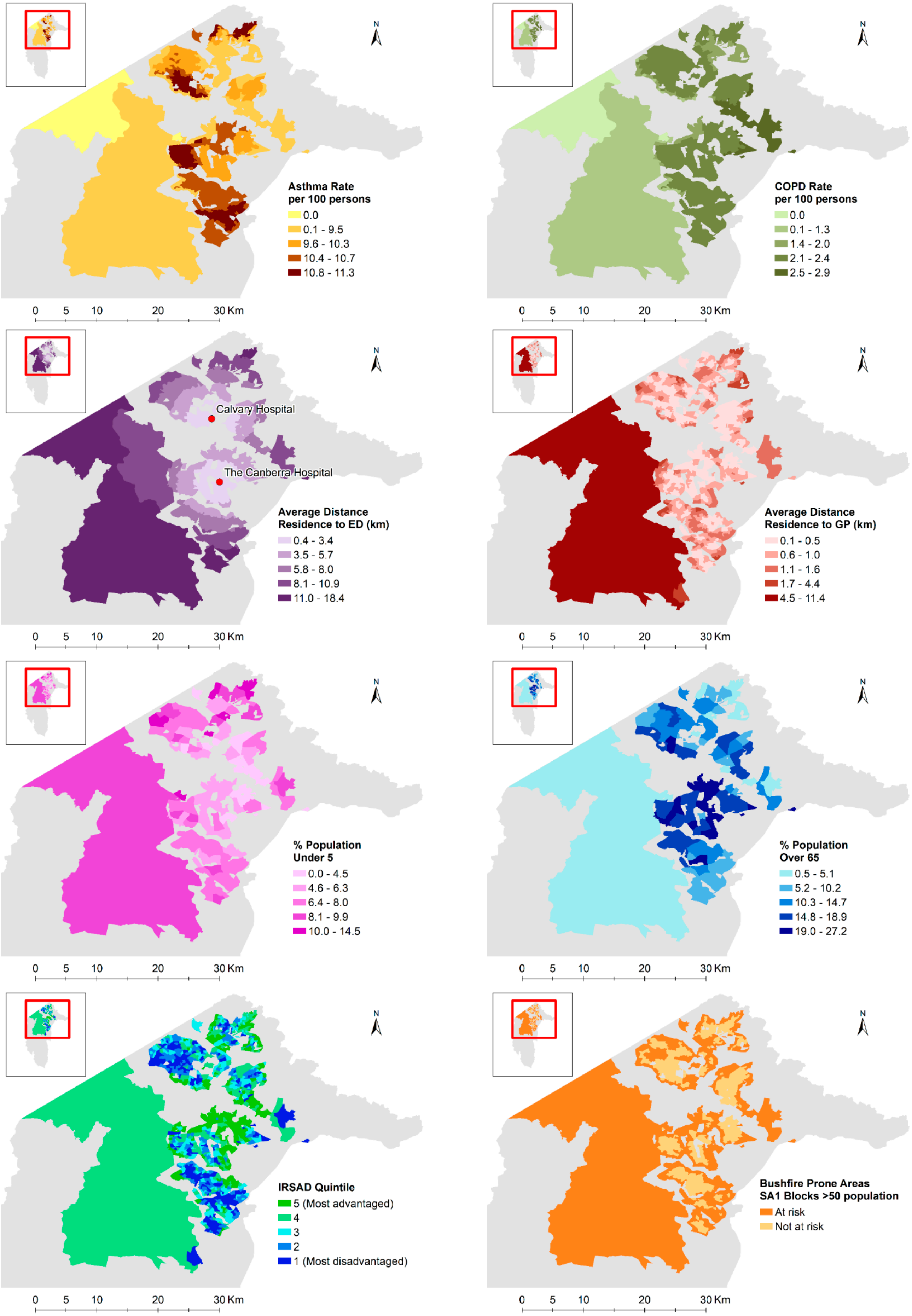

Figure S2: Histograms of ED distance and natural log transformed ED distance

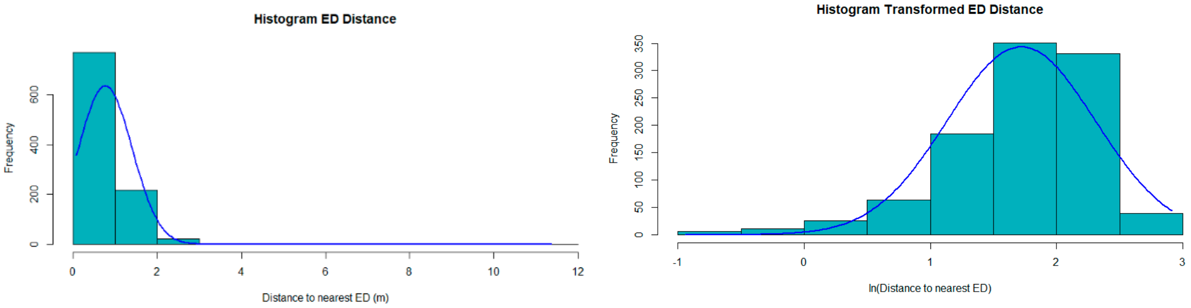

Figure S3: Histograms of GP distance and natural log transformed GP distance

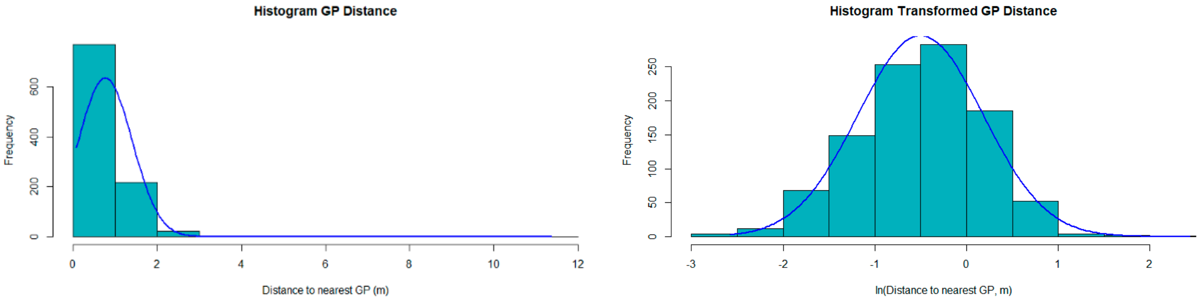

TableS1: Cluster analysis results all variables: Moran's Index and Getis-Ord General G Statistic

Variable	Moran Index	z	p	Observed General G	z	p	Threshold Distance (m)	No. Neighbours		
								Min	Average	Max
Asthma Rate	0.61	59.6	0.0000	0.000024	4.5	0.0000	1,830	8	23.3	44
COPD Rate	0.62	51.3	0.0000	0.000019	5.2	0.0000	1,500	8	16.3	35
ED	0.70	170.5	0.0000	0.000074	18.5	0.0000	7,220	8	205.2	348
GP	0.37	38.7	0.0000	0.000028	9.4	0.0000	1,890	8	24.7	46
Under5	0.97	79.8	0.0000	0.000022	21.2	0.0000	1,500	8	16.3	35
Over65	0.88	72.7	0.0000	0.000020	2.9	0.0036	1,500	8	16.3	35
IRSAD	0.28	27.4	0.0000	0.000024	-4.2	0.0000	1,900	8	24.9	46

Figure S4: Geographically Weighted Poisson Regression Local Coefficients for Asthma

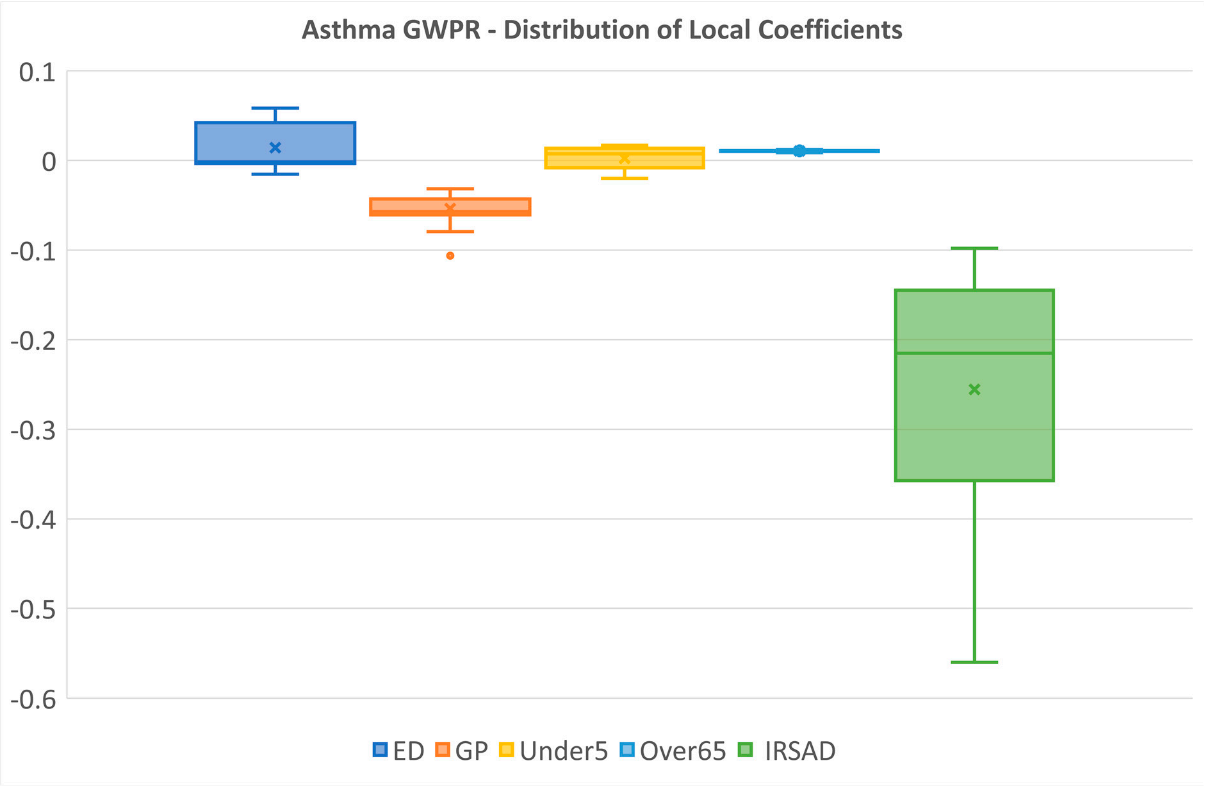

Figure S5: Geographically Weighted Poisson Regression Local Coefficients for COPD

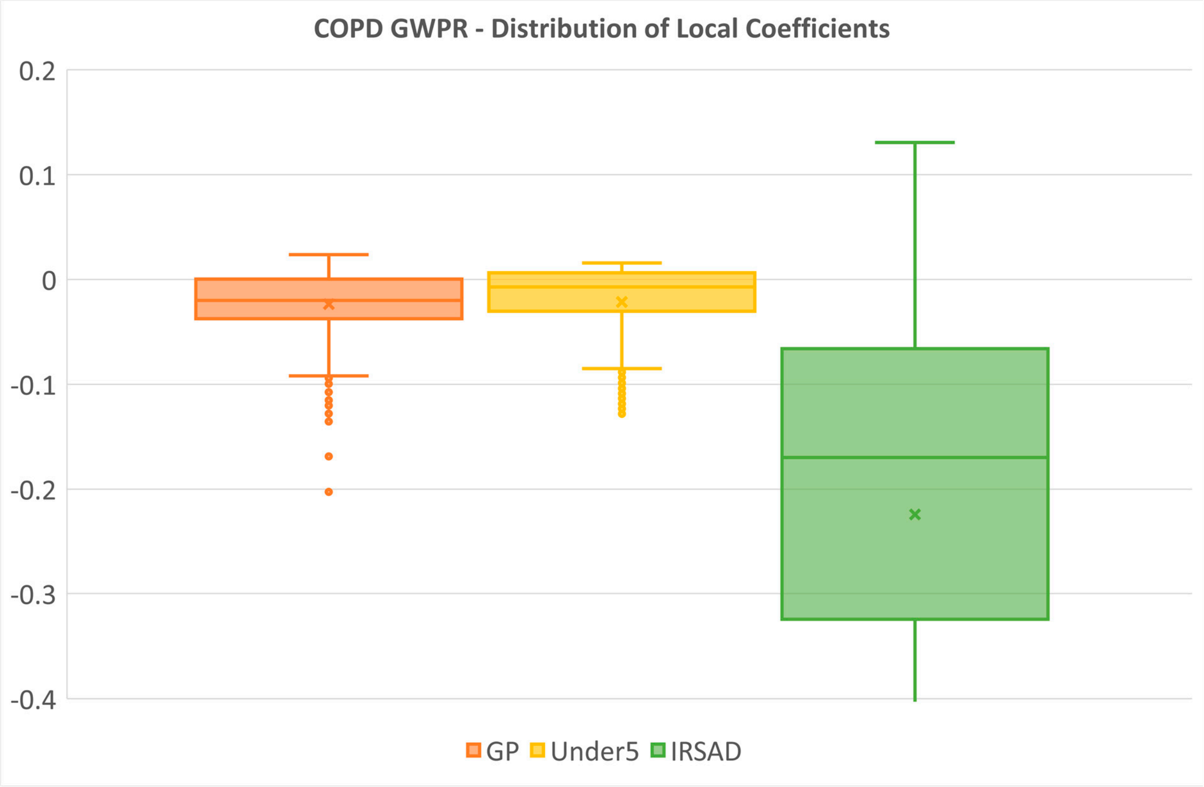

Supplement: Supplementary file 1 [file ijerph-17-06396-s001.pdf]
